# Low‐level germline mosaicism of a novel *SMARCA2* missense variant: Expanding the phenotypic spectrum and mode of genetic transmission

**DOI:** 10.1002/mgg3.1763

**Published:** 2021-07-22

**Authors:** Nina Pan, Songchang Chen, Xiaoqiang Cai, Jianli Li, Tao Yu, He‐feng Huang, Jinglan Zhang, Chenming Xu

**Affiliations:** ^1^ International Peace Maternity and Child Health Hospital, School of Medicine Shanghai Jiao Tong University Shanghai China; ^2^ Shanghai Key Laboratory of Embryo Original Diseases Shanghai China; ^3^ Obstetrics and Gynecology Hospital Institute of Reproduction and Development Fudan University Shanghai China; ^4^ State Key Laboratory of Genetic Engineering and MOE Engineering Research Center of Gene Technology, School of Life Sciences Fudan University Shanghai China; ^5^ Beijing BioBiggen Technology Co., Ltd., Beijing China; ^6^ Clinical Research Center for Birth Defects and Rare Diseases School of Medicine Shanghai Jiao Tong University Shanghai China; ^7^ Key Laboratory of Reproductive Genetics Ministry of Education Zhejiang University Hangzhou China; ^8^ Women's Hospital School of Medicine Zhejiang University Hangzhou China

**Keywords:** amplicon sequencing, germline mosaicism, Nicolaides–Baraitser syndrome, QLQ domain, *SMARCA2* gene

## Abstract

**Background:**

Nicolaides–Baraitser syndrome (NCBRS) is a severe neurodevelopmental disorder with multiple abnormalities. To date, all pathogenic variants in *SMARCA2* causing NCBRS are *de novo* and most are missense variants located in the ATPase domain of SMARCA2 protein.

**Methods:**

In this study, a familial trio whole‐exome sequencing was performed on the proband presenting with intellectual disability, early‐onset epilepsy, and autistic features. A novel missense variant c.553C>G (p.Gln185Glu) in *SMARCA2* was identified, which is located in the QLQ domain. The same variant was subsequently also found in the mother's ongoing pregnancy. Samples from accessible tissues such as saliva and sperm other than blood were collected from the parents, and the detection of the target variant was performed by amplicon‐based deep sequencing.

**Results:**

Low‐level mosaicism of the target variant c.553C>G (p.Gln185Glu) was detected in the father's sperm with allele fraction of 2.8% by amplicon‐based deep sequencing, which was not detected in either parents’ blood or saliva specimens. Heterozygosity of this variant was confirmed in the proband.

**Conclusion:**

This is the first report of paternal germline mosaicism for a *SMARCA2* disease‐causing variant. In addition, the missense variant c.553C>G (p.Gln185Glu) in the QLQ domain causes mainly neurological and developmental phenotypes with unremarkable characteristic facial features and limb abnormalities. Our findings expand the phenotypic spectrum and mode of genetic transmission associated with the *SMARCA2* variants.

## Graphical abstract

A novel SMARCA2 missense variant in the proband was found due to low‐level paternal germline mosaicism as the first disease‐causing variant in the QLQ domain associated with solely neurological and developmental phenotypes of NCBRS, which provides new insights into the structure–function relationship of this subunit in the SWI/SNF complexes.

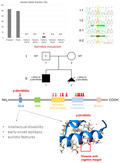

## 1 INTRODUCTION

Nicolaides–Baraitser syndrome (NCBRS) is an autosomal dominant disease with multiple anomalies characterized by distinctive facial features, abnormalities of trunk and limbs, seizures, severe intellectual disability, and impaired language development (Nicolaides & Baraitser, 1993; Sousa et al., 2009). Seizures often begin in infancy which is difficult to be managed with antiepileptic drugs (Sousa et al., 2009). The signs and symptoms are highly variable in affected individuals. NCBRS is rare and fewer than 100 cases have been reported (Sousa & Hennekam, 2014).

Pathogenic variants in *SMARCA2* (OMIM number: 600014) were identified as the underlying genetic causes of NCBRS (Van Houdt et al., 2012). *SMARCA2* is located on chromosome 9p24.3, and it encodes a component of SWI/SNF complexes, which play an important role in chromatin remodeling and regulation of downstream gene expression in neural development (Sokpor et al., 2017). To date, *de novo* pathogenic variants were previously reported in all affected individuals with NCBRS (Bramswig et al., 2015; Ejaz et al., 2016; Hofmeister et al., 2020; Hu et al., 2019; Ma et al., 2020; Mari et al., 2015; Sethi et al., 2019; Sousa & Hennekam, 2014; Tang et al., 2017). Nearly all disease‐causing variants are missense changes and clustered in the highly conserved ATPase domain, which is critical for ATP‐binding/hydrolysis and DNA‐binding in transcriptional regulation (Bogershausen & Wollnik, 2018).

Here, we report a family in which the disease‐causing *SMARCA2* variant in the proband was found due to low‐level paternal germline mosaicism. To our knowledge, this is the first case report of germline mosaicism for *SMARCA2*‐related disease with a confirmed molecular finding. We also report it as the first disease‐causing variant in the QLQ domain associated with solely neurological and developmental phenotypes of NCBRS, which provides new insights into the structure–function relationship of this subunit in the SWI/SNF complexes.

## 2 MATERIALS AND METHODS

### 2.1 Methods

Peripheral blood and saliva samples were collected from both parents, and a semen sample was collected from the father as well. Peripheral blood and amniocytes were collected from the proband and the current pregnancy, respectively. Genomic DNA was extracted using commercial kits according to the manufacturer's instructions (Qiagen, Germany). Amplicon‐based deep sequencing was used for mosaicism detection and the estimation of variant allele fraction. Briefly, the genomic region containing the missense *SMARCA2* (reference sequence: NG_032162.2, transcript: NM_003070.5) variant was targeted and amplified using specific primers (forward: 5‐CCAACAGAGGTCCCTCACCT‐3, reverse: 5‐GCCTCGGGCCAGCATTTTAT‐3) with PCR conditions as following: (1) initiation denaturation at 98℃ for 2 min, (2) 98℃ for 10 s, 70℃ (−1.0℃/cycle) for 30 s, 72℃ for 30 s, 10 cycles, (3) 72℃ for 5 min. Universal sequencing primers were added by the second round of PCR and amplicons were generated with different barcodes for each sample. Multiplexed sequencing was run on the MGISEQ‐2000 sequencer (MGI Tech Co., Ltd., China). Sequencing data were analyzed by an in‐house developed bioinformatics pipeline. After alignment to the human reference genome (GRCh38) and removal of duplicate reads, the target variant allele fraction was calculated for each sample. DNA from the proband was used as positive control and NA12878 DNA (Coriell Institute) was used as a negative control. Background noise and limit of detection were determined using negative control and a serial dilution (from 0.01% to 10%) of positive control.

## 3 RESULTS

### 3.1 Clinical presentation

The proband is an 8‐year‐old Chinese boy who presented with intellectual disability, recurrent, and intractable epilepsy since 2 years after birth. The proband was born to a 26‐year‐old, G2P0 mother by spontaneous vaginal delivery at term, without pregnancy or birth complications. Developmental delay was noticed when he was about 1 year old, especially on verbal and intellectual development, and the fine motor control and coordination of movement were below average. At 2 years old, the first episode of seizures occurred during sleep, which lasted 1 to 2 min and was associated with jerking of bilateral upper limbs. The seizure was recurrently followed by the frequency increased gradually from ~1/month to ~1/day. At the age of four, seizures started to also occur in the awake period, with the manifestation of fear, startle, and jerking of upper limbs. At the age of seven, the proband manifested intellectual disability, poor verbal expression, and features of hyperactivity disorder, with seizures occurred one to three times per day. The electroencephalogram (EEG) showed abnormal waves at the age of three and seven. History of febrile seizure, brain infection or head trauma was denied, and there is no family history of epilepsy. The administration of antiepileptic drugs such as oxcarbazepine and clobazam showed partial relief on seizure symptoms and frequency. The proband was also evaluated at the autism clinic at age four, with records of limited social communication and repetitive patterns of behavior. No facial dysmorphic features were noticed during the evaluation.

### 3.2 Genetic testing results

**FIGURE 1:**
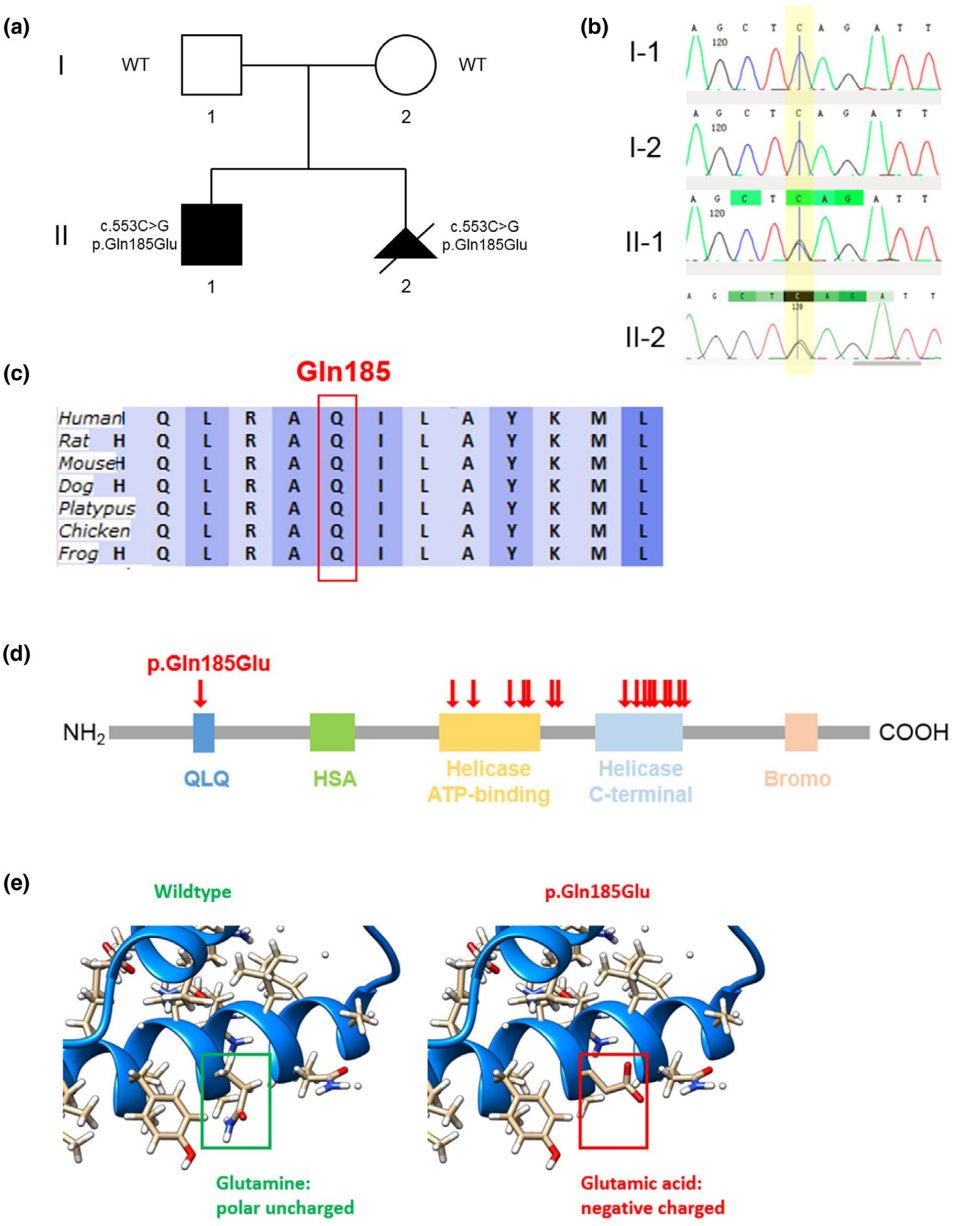
Family pedigree with genotype results for the p.Gln185Glu variant and its characterization. (a) Pedigree of the family in this study with genotype results for the p.Gln185Glu variant. WT, wildtype. (b) Sanger sequencing chromatograms of the *SMARCA2* c.553C>G (NM_003070.5) variant. The homozygosity of cytosine and heterozygosity of cytosine/guanosine are highlighted for I‐1/I‐2 and II‐1/II‐2, respectively. (c) Sequence conservations of glutamine residue at codon 185 within the SMARCA2 protein in different species. (d) A schematic representation of the SMARCA2 protein and the variant p.Gln185Glu was identified in this study. Red arrows indicate the distribution of previously reported pathogenic variants in the ClinVar database. QLQ, Gln‐Leu‐Gln domain; HSA, small helicase/SANT‐associated domain; Bromo, bromodomain. (e) Structural change in the motif caused by the replacement of glutamine by glutamic acid

The proband underwent karyotyping and chromosomal microarray analysis (Affymetrix CytoScan 750K array), and no chromosomal abnormalities were detected. A familial trio clinical exome sequencing was performed in which a *de novo* missense variant in *SMARCA2* (NM_003070.5: c.553C>G, p.Gln185Glu) was found in the proband. No other pathogenic or likely pathogenic variants related to the proband's phenotype were identified. Both parents were phenotypically normal and were tested negative for this variant by whole‐exome and Sanger sequencing (Figure 1a,b). The c.553C>G (p.Gln185Glu) variant was neither reported in literature or human population databases such as gnomAD. It was predicted as disease‐causing by multiple computational tools including PolyPhen‐2, SIFT, and MutationTaster, with a REVEL score of 0.831 (Ioannidis et al., 2016). No splicing effects were predicted by *in silico* tools of splice‐site alterations. Based on its mode of inheritance and genotype–phenotype correlation, this variant was classified as likely pathogenic according to ACMG guidelines since the criteria of PS2, PM2, PP2, and PP3 were met in this case (Richards et al., 2015). Glutamine residue at codon 185 is highly conserved across different species (Figure 1c), and it is located in the QLQ domain within SMARCA2 protein (Figure 1d). It should be noted that the majority of previously reported *SMARCA2* pathogenic variants in the ClinVar database were distributed in the Helicase ATP‐binding domain and Helicase C‐terminal domain (Figure 1d), and the p.Gln185Glu variant was the first disease‐causing variant identified in the QLQ domain, which resulted in the replacement of polar uncharged glutamine by negatively charged glutamic acid, and disturbance of the chemical and structural properties of SMARCA2 protein (Figure 1e).

The proband was then diagnosed with NCBRS based on phenotypic and molecular findings. The proband's mother had an ongoing pregnancy at a gestational age of 9 weeks. Prenatal diagnosis was offered to the family for the fetus after the *SMARCA2* variant was identified in the proband. By amniocentesis, it was confirmed that the same variant c.553C>G (p.Gln185Glu) was present in the fetus (Figure 1a,b). The pregnancy was terminated after genetic counseling and the presence of germline mosaicism was suspected. Both parents were referred for further genetic testing.

### 3.3 The identification of germline mosaicism

**FIGURE 2:**
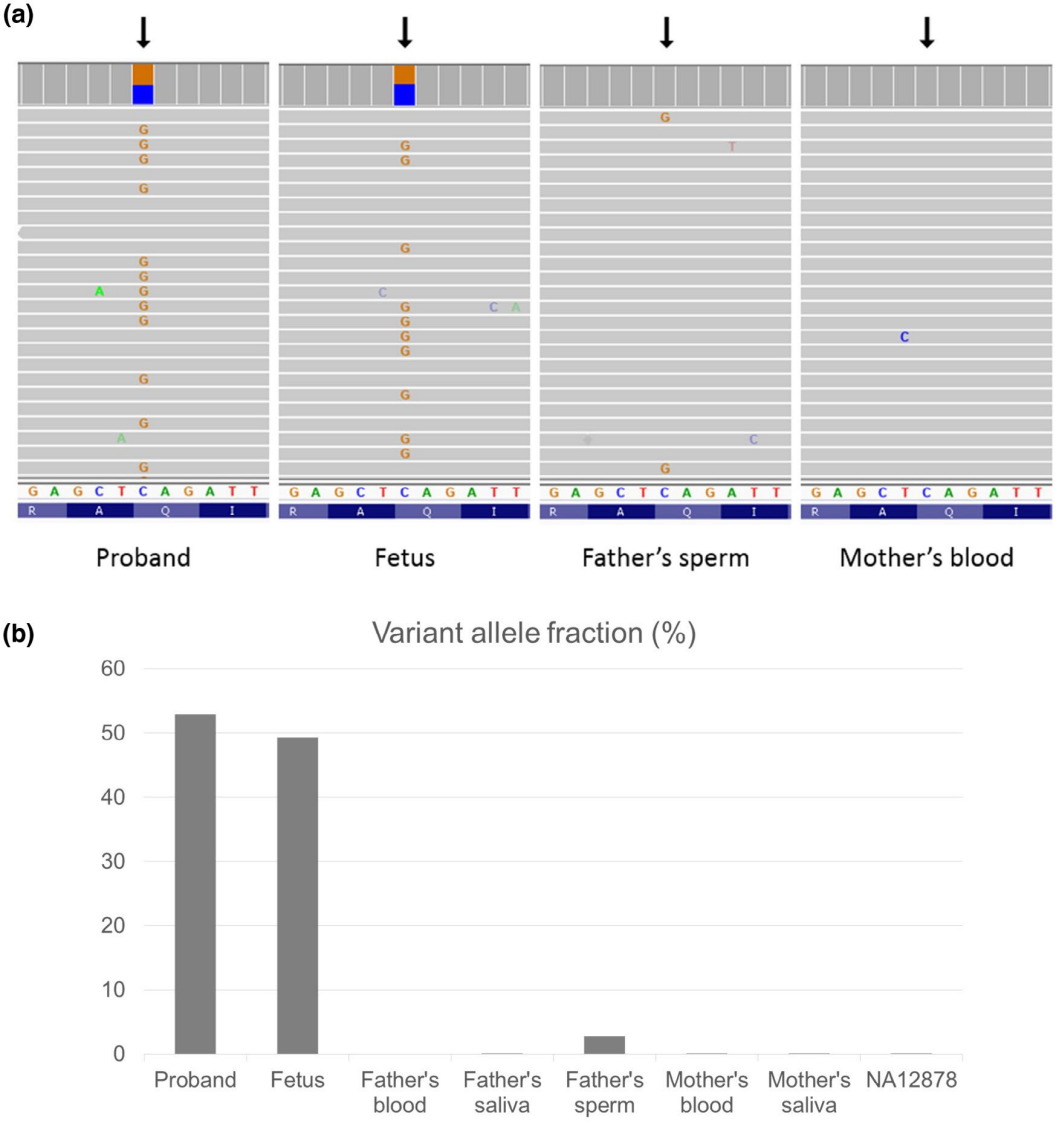
Presence and variant allele fraction of *SMARCA2*: c.553C>G p.Gln185Glu variant detected by amplicon‐based deep sequencing. (a) Amplicon sequencing data of representative samples shown in Integrative Genomics Viewer. Arrows indicate the base position of c.553C>G (NM_003070.5). (b) Variant allele fraction determined by sequencing reads of all tested samples

Amplicon‐based deep next‐generation sequencing (NGS) was performed on samples such as blood and saliva collected from both parents, and sperm collected from the father. DNA isolated from the proband and his fetal sibling were included as positive controls. The NGS coverage of the target locus *SMARCA2* c.553C>G (p.Gln185Glu) was more than 5000X in all samples tested. Nearly half of the total reads harbored the variant G allele in the PCR products amplified from the proband's and the fetus’ DNA. A low percentage of sequencing reads with the variant G allele were found in the father's sperm DNA, and no such reads were seen in the mother's blood (Figure 2a). The variant allele fraction calculated from sequencing reads indicated heterozygosity of C/G on this position in the proband (52.9%) and the fetus (49.3%). About 2.8% reads of pathogenic allele G were detected in the father's sperm, suggesting the presence of low‐level germline mosaicism (Figure 2b). No pathogenic allele was detected in blood or saliva samples from both parents when comparing with the background noise (0.16%) determined by negative control.

## 4 DISCUSSION

In this report, we described the first case where paternal germline mosaicism of a *SMARCA2* disease‐causing variant was the underlying cause of NCBRS. This disease is considered genetically homogeneous as all patients were found to have *de novo* pathogenic variants in *SMARCA2* (Sousa & Hennekam, 2014). Our finding in this study suggests that germline mosaicism needs to be ruled out in families affected by NCBRS for better planning of future pregnancies. When preimplantation genetic testing is possible, the identification of potential parental germline mosaicism will facilitate the implantation of an unaffected embryo. Prenatal diagnosis for such families is always useful regardless of the conceiving strategy. It is possible that germline mosaicism is present in previously documented NCBRS patients and families, most of which were diagnosed based on exome sequencing results. The limited depth of coverage and sample types (mostly leukocytes) may not be able to reveal the presence of pathogenic variants in germline cells. It may be worth evaluating previously reported NCBRS families in order to obtain the frequency of occurrence of germline mosaicism.

Germline mosaicism is a relatively common underlying mechanism in families who have more than one affected offspring affected by *de novo* dominant variants born to phenotypically normal parents. It is due to spontaneous pathogenic variants which occur predominately during mitotic or meiotic divisions in oogenesis and spermatogenesis (Spinner & Conlin, 2014). Because of its easier accessibility, studies of mosaicism on sperms are far more than eggs. Sperm mosaicism has been reported in patients with various genetic disorders (Breuss et al., 2020; Pauli et al., 2009; Yang et al., 2017), and the pathogenic allele fractions vary from very low (~0.03%) to moderate level (~39%) (Yang et al., 2017). In our case, the mosaicism appeared to be restricted to the gonadal organ because no pathogenic alleles were detected in other somatic cells such as father's leukocytes or buccal cells. It is also possible that the level of somatic mosaicism was lower than the limit of detection (0.5%). Given the low‐level mosaicism (<3%) and two successive offsprings with the same variant in this family, it is of interest to explore if the sperms with the *SMARCA2* variant possess increased advantage during competition with sperms containing the normal allele.

The SMARCA2 protein is a subunit of the SWI/SNF complexes which participate in chromatin remodeling in an ATPase‐dependent manner. In a review of 61 patients with NCBRS, the vast majority (n = 59) of pathogenic variants are missense and only a few (n = 2) are in‐frame deletion (Sousa & Hennekam, 2014). All previously reported pathogenic variants are located through exon 15 to exon 25, which are within the ATPase domain. It is hypothesized that the dominant‐negative or gain‐of‐function effect, but not haploinsufficiency, underlies the role of mutated *SMARCA2* in NCBRS (Sousa & Hennekam, 2014; Van Houdt et al., 2012). Herein, we reported the first disease‐causing variant in the QLQ domain of SMARCA2 protein. The QLQ domain is featured by the conserved Gln‐Leu‐Gln residues. For the SMARCA2 protein with Gln185Glu variant, the chemical and structural properties may be severely disrupted by replacing polar uncharged glutamine with negatively charged glutamic acid, but its effects on complexes assembly or ATPase activity are currently unknown. The mutant SMARCA2 protein causes dysregulation of the expression of neural progenitor genes and neural enhancer reprogramming, and genome‐wide DNA methylation changes are also relevant to NCBRS pathophysiology (Chater‐Diehl et al., 2019; Gao et al., 2019). It should be noted that the *SMARCA2* RNA is highly expressed in male testis. Assessment of the *SMARCA2* variant c.553C>G p.Gln185Glu in neural developmental signaling pathways, DNA methylation signatures, and spermatocytes growth would be of interest in further studies.

In conclusion, this is the first reported germline mosaicism case of *SMARCA2*‐related disease. The inheritance mode, tissue of origin, and level of mosaicism were determined by deep sequencing. It suggests that genetic counseling and recurrence risk estimation could benefit from the germline mosaicism assessment. This is also the first reported disease‐causing variant located in the QLQ domain of the SMARCA2 protein associated with intellectual disability, early‐onset epilepsy, and autistic features, suggesting the importance of the high conservation and functional role of this motif.

## CONFLICT OF INTERESTS

XC, JL, TY, and JZ are employees or shareholders of Beijing BioBiggen Technology Co., Ltd. The other authors declare no conflict of interest.

## AUTHOR CONTRIBUTIONS

NP, XC, JZ, and CX conceived and designed the study. SC, HH, and CX performed clinical assessments on patients and provided specimens. NP, XC, JL, and TY conducted the experiments and analyzed the data. NP, XC, and JZ wrote the manuscript. JZ and CX supervised the study.

## ETHICAL COMPLIANCE

This study was approved by the institutional review boards of the International Peace Maternity and Child Health Hospital (GKLW2019‐52) and the Obstetrics and Gynecology Hospital of Fudan University (2020‐178). The signed informed consent was obtained from patients.

## Data Availability

The data in this study are available from the corresponding author upon reasonable request.
